# Multivalent vaccines for invasive *Salmonella* disease: need, rationale, and immunological foundations

**DOI:** 10.1128/iai.00118-25

**Published:** 2026-01-06

**Authors:** Pietro Mastroeni, Omar Rossi, Francesca Micoli

**Affiliations:** 1 University of Cambridge 2152, Cambridge, United Kingdom; 2 GSK, Vaccines Institute for Global Health (GVGH) 622628 , Siena, Italy; University of California at Santa Cruz, Santa Cruz, California, USA

**Keywords:** *Salmonella*, vaccines

## Abstract

*Salmonella enterica* infections are a major cause of morbidity and mortality worldwide, especially in sub-Saharan Africa and in the Asian continent, and are increasingly associated with antimicrobial resistance. *Salmonella enterica* serovars Typhi and Paratyphi A, B, and C cause enteric fever, while non-typhoidal *Salmonella* serovars (usually Typhimurium and Enteritidis) cause mainly gastroenteritis which can lead to systemic infections. Vaccines are only licensed against *S*. Typhi, but different combinations are in clinical development to prevent *S*. Typhi and *S*. Paratyphi A or *S*. Typhi and non-typhoidal *Salmonella*. Here, we describe elements of the pathogenesis of and immunity to *Salmonella* that are critical to guide the rational design of vaccines. We highlight how the choice of appropriate immunogenic and protective antigens would be essential to achieve the maximum coverage of serovars in a multivalent *Salmonella* vaccine. The principal vaccines under development at the preclinical and clinical stages are described, together with considerations on the technical and clinical feasibility of moving combination vaccines toward licensure.

## INTRODUCTION

Invasive serovars of the Gram-negative enterobacterium *Salmonella enterica* are a significant cause of morbidity and mortality worldwide. *Salmonella enterica* serovar Typhi (*S*. Typhi) and serovar Paratyphi (*S*. Paratyphi) are the causative agents of enteric fevers. The incidence of enteric fevers remains high despite a decline in recent years from about 21 million cases and over 200,000 deaths in 2000 to 9.3 million cases and 107,000 deaths in 2021 (1–3). Non-typhoidal *Salmonella* (NTS), especially lineages of serovars Typhimurium sequence type (ST) 313 and Enteritidis ST11, causes acute systemic infections with bacteriaemia (invasive non-typhoidal *Salmonella* disease—iNTS). Young age (<3 years old), often with concomitant malaria, anemia, or malnutrition, as well as acquired (e.g., HIV) or genetic immune deficiencies, is a significant predisposing factor for iNTS (4–7). Despite recent progress in reducing the global burden of iNTS, the disease remains a serious public health concern: in 2021, an estimated 510,000 cases of iNTS were recorded, resulting in approximately 62,000 deaths (8). While these figures represent a substantial improvement compared to earlier estimates—such as the over 3 million cases and approximately 680,000 deaths reported in 2010 (9)—they underscore the continued impact of iNTS on vulnerable populations worldwide. iNTS disease has high mortality (approximately 15%) due to many complications, with septicemia and anemia as the principal ones (10). Enteric fevers are prevalent in Southeast Asia, and iNTS have a higher incidence in sub-Saharan Africa; however, there is considerable overlap between the two diseases that are broadly present in both geographical areas (11, 12).

Improved sanitation and the reduction of predisposing factors have greatly impacted the reduction in the global incidence and mortality of enteric fevers and iNTS disease. However, these diseases are still a threat for a large part of sub-Saharan Africa and the Asian continent, especially in the light of increasing antimicrobial resistance (AMR) (13–15). Vaccines remain key components of our toolbox in the fight against bacterial infections, especially those such as systemic salmonellosis that show high and increasing levels of AMR (16–19).

Current licensed vaccines against *S*. Typhi infections include live attenuated strains (Ty21a) and several preparations based on the Vi surface polysaccharide antigen, alone or conjugated to protein carriers. Several vaccines against iNTS disease and paratyphoid fever are currently at different stages of development and testing. These include the use of surface polysaccharide antigens conjugated to protein carriers and bacterial outer membrane vesicles (OMVs) (20).

The overlapping geographic incidence of enteric fevers and iNTS diseases (11, 12), the variety of serovars that cause these infections (e.g., Typhi, Paratyphi, Typhimurium, Enteritidis), the documented presence of “atypical” iNTS serovars (21), and the possible epidemiological emergence of new serovars are steering current research and development efforts toward multivalent vaccines. These have the potential to protect against a multitude of serovars and could be easily adapted to include additional antigens from new epidemiologically relevant *Salmonella* serovars. Furthermore, multivalent vaccines will substantially contribute to AMR reduction, increase commercial attractiveness through reduced cost of goods, improve acceptance among end users and healthcare providers, and enhance adherence to vaccination schedules, ultimately leading to higher and more equitable vaccination coverage.

## UNDERSTANDING THE PATHOGENS TO DESIGN BETTER VACCINES

To target a pathogen with vaccine-induced immune responses, it is essential to understand how it behaves within the host. The complex behavior of *Salmonella* within host tissues has been unraveled by studies in preclinical murine models where multiple parameters—such as localization, patterns of bacterial spread, and the effects of immunity and vaccination on the disease process—can be accurately monitored. These models have been invaluable for understanding the pathogenesis and immunity to *Salmonella* and for guiding the rational design of vaccines and therapeutic approaches.

After ingestion, *Salmonella* invades the gut with the contribution of the *Salmonella* Type 3 secretion system encoded by the *Salmonella* pathogenicity island 1 (SPI1) (22) to reach the mesenteric lymph nodes, and then disseminates via the blood to an intracellular location, mainly within resident phagocytes of the spleen, liver, and bone marrow (23–27). Multicellular pathological lesions consisting of clusters of phagocytes form at the initial sites of infection, with the involvement of adhesion molecules and complex cytokine networks (25, 27–31). The escalation of the cell-mediated immune response restrains bacterial growth within the infected cells (32–38). However, a key feature of systemic *Salmonella* infections is their dispersiveness; the intracellular multiplication of the bacteria is paralleled by the increase in the number of infectious foci in the infected tissues. This is due to *Salmonella* escaping from established foci of infection to reach distant sites in the body via the extracellular space; once these new sites are reached, the bacteria can home again intracellularly (27, 39–41). Therefore, the within-host life cycle of *Salmonella* has two distinct phases: an intracellular phase of replication and an extracellular phase of cell-to-cell spread. This has crucial implications for understanding the mechanisms and correlates of protective immunity and for informing vaccine design. In fact, control of intracellular replication is likely to require cell-mediated immunity with the involvement of T cells and cytokines. Conversely, during their extracellular phase of spread, the bacteria can be targeted by antibodies that can kill them possibly via opsono-phagocytosis and serum bactericidal activity, with the involvement of Fc receptors and/or complement (35, 42–45). This biphasic model of *Salmonella* growth and spread is consistent with the requirement for both antibodies and T cells observed in vaccine-mediated protection against virulent *Salmonella* in mice (46).

Evidence from humans also supports a dual role for antibodies and cell-mediated immunity (T cells/phagocytes) in vaccine-induced protection against *Salmonella*. For example, peak incidence of iNTS infection, observed in young children between 6 and 18 months of age, coincides with the lack of antibody-dependent bacterial killing activity in their sera (4, 47, 48). Antibodies and complement have been shown to be essential for the oxidative burst and killing of *Salmonella* by blood cells in Africans (49). Vaccines that contain the *Salmonella* Vi surface polysaccharide and therefore do not contain *Salmonella*-specific T cell antigens mainly induce antibodies and still offer good protection against typhoid fever (50). A role for cell-mediated immunity in resistance to systemic salmonellosis in humans is exemplified by the high incidence of iNTS observed in HIV-infected individuals and in children with malaria, where T cell and phagocyte antimicrobial functions are severely reduced, respectively (4).

## WHICH PLATFORMS ARE BEST FOR THE DEVELOPMENT OF MULTIVALENT *SALMONELLA* VACCINES? LIVE ATTENUATED VACCINES VERSUS NON-LIVING PREPARATIONS

Safety, immunogenicity, protective ability, ease of production, adaptability, and reduced costs are some of the key issues that need to be carefully considered for future development and optimization of broadly protective multivalent *Salmonella* vaccines.

Live attenuated vaccines would be ideal platforms due to their ability to induce both protective T cell–mediated immunity and antibody responses, as well as their ease of distribution. These vaccines elicit local mucosal and systemic immunity and can be engineered to express multiple antigens from different pathogens. However, many live attenuated *Salmonella* vaccine candidates may pose potential safety risks, as they can retain excessive virulence and can kill severely immune-deficient animals in preclinical studies (51–54). Immune-suppressive conditions that could potentially enhance the virulence of live *Salmonella* (e.g., HIV, malaria, malnutrition, anemia) have a high incidence in those very same areas where the vaccines are most needed. Therefore, more work needs to be done to establish whether live attenuated *Salmonella* mutants, similar to the ones that are unsafe in severely immune-deficient mice, could also pose risks for immunosuppressed vaccinees (4).

**Fig 1:**
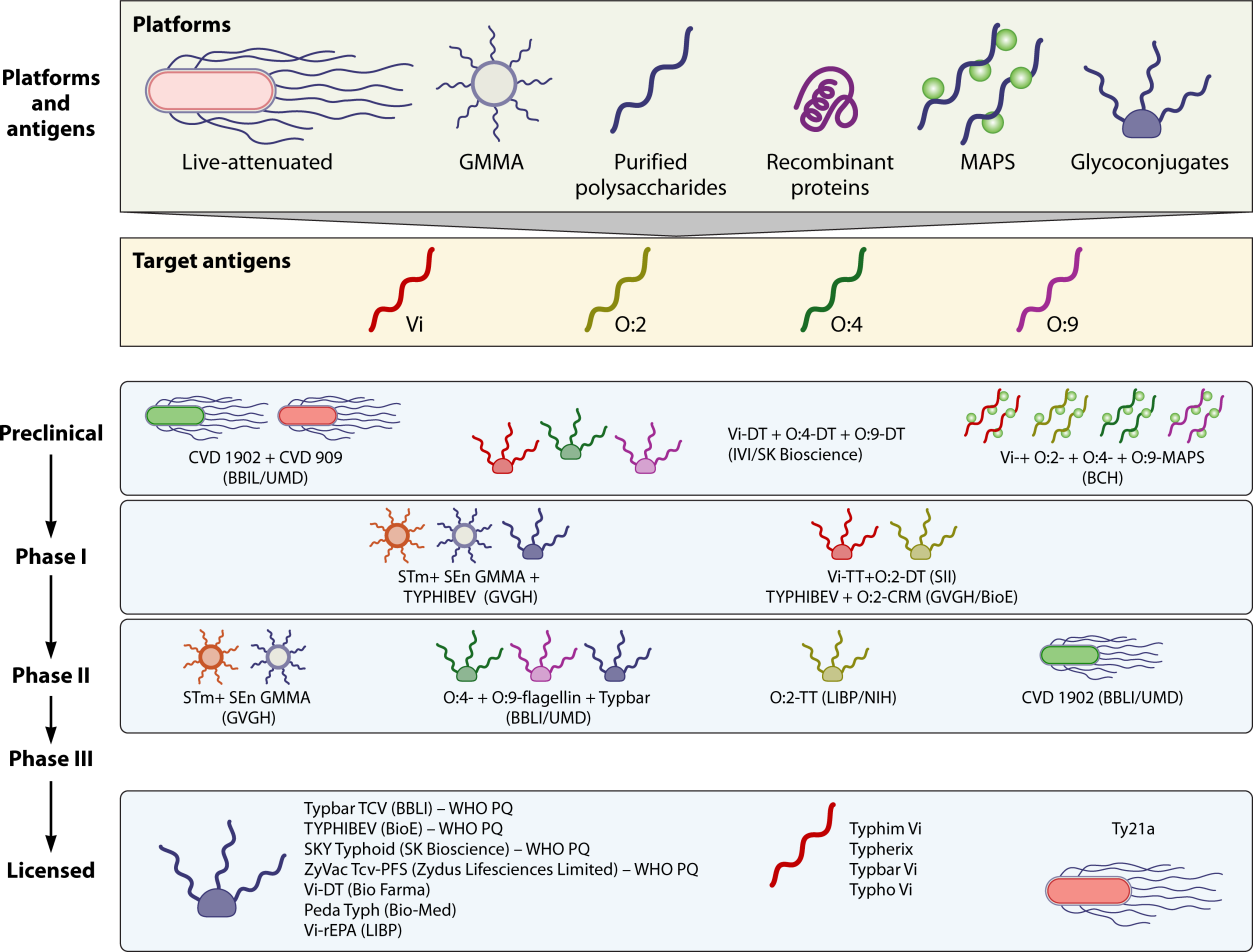
Different platforms in use for presentation of *Salmonella* antigens and main vaccines at different stages of development.

Many of the vaccine platforms that are currently being developed for the prevention of enteric fevers and iNTS are based on non-living preparations. These mostly include surface polysaccharides (e.g., Vi and O-antigens), usually conjugated with protein carriers (glycoconjugates), vesicles from the bacterial outer membrane (OMV), surface proteins, and combinations thereof (20) (Fig. 1).

The first type of non-living vaccine against *S*. Typhi consisted of the Vi polysaccharide alone. Its limited effectiveness in younger children can be attributed to the immune response elicited by pure polysaccharide vaccines, which lack T cell involvement. The absence of T cell help results in several immunological limitations, including inability to induce immunological memory, a short duration of the antibody response, and hypo-responsiveness to subsequent vaccinations. Glycoconjugation consists of chemical linkage of the polysaccharide to an appropriate carrier protein. Chemically detoxified tetanus and diphtheria toxins as well as the genetically modified diphtheria toxoid CRM_197_ are among the most used carriers (55). The protein is needed to provide T cell help, thus producing class switching to IgG, generating memory responses, and making the vaccines effective in young children. Glycoconjugation is a well-established technology, with effective glycoconjugate vaccines licensed worldwide against many pathogens, including *Haemophilus influenzae* type b, *Neisseria meningitidis* serogroups A, C, and ACWY, *Streptococcus pneumoniae*, and *S*. Typhi (56).

More recently, Multiple Antigen Presenting System (MAPS) technology has been proposed as an alternative for Vi and O-antigens delivery. This platform utilizes high-affinity, non-covalent interactions between biotin-tagged polysaccharides and rhizavidine-fused proteins, potentially allowing for broader immune responses by combining polysaccharides with conserved pathogen-specific proteins. The resulting constructs mimic features of a whole cell construct for B- and T-cell activation (57).

OMVs have also been proposed for the development of *Salmonella* vaccines. OMVs present multiple antigens (e.g., polysaccharides and proteins) to the immune system in their native membrane environment and possess lipoproteins and lipopolysaccharides on the surface that exert an immune stimulatory effect. Genetic manipulation is used to reduce lipopolysaccharide-mediated reactogenicity, usually by reducing the number of lipid A acyl chains (58), and can be used to display and/or overexpress homologous or heterologous antigens (proteins or saccharides) and to delete unwanted interfering antigens (59).

Evidence from preclinical studies shows that non-living vaccines are far less protective than live attenuated ones, and this inferiority is ascribed to their inability to elicit “protective” T cell responses (42). Antibodies, in the absence of concomitant T cell immunity, appear to increase the killing of *Salmonella* soon after infection but have no effect on bacterial growth and, surprisingly, on bacteremia (42, 60). So, how can we expect non-living vaccines to protect, and how do we explain the efficacy of currently used ones? It is likely that non-living vaccines confer protection either by preventing the early onset of the infection or by reducing the bacterial load in the early stages of the disease, thus allowing T cell immunity to develop before the infection escalates to high bacterial burdens in the tissues. Conversely, in individuals where background T cell memory is already present, a non-living vaccine would produce *Salmonella*-specific antibody responses that, in addition to T cells, would yield an overall highly protective immune response. This reasoning is supported by observations on the relationship between immunity and disease incidence in Africa. In fact, children in Malawi develop T cell immunity to *Salmonella* in the first few months of their life, but they do not acquire resistance to NTS until later, when they also produce *Salmonella*-specific antibodies (48). Vi-polysaccharide typhoid vaccines are more protective in field studies in endemic areas than in human volunteers participating in controlled vaccination and challenge studies, further suggesting that an immunological background due to previous exposure to cross-reactive antigens may affect vaccine efficacy (61–63).

These observations raise the possibility that different vaccines may be needed for travelers who do not already have an immunological background and for vaccinees in endemic areas, where low-grade exposure to *Salmonella* or to other bacteria displaying cross-reactive antigens is probably more common. Furthermore, it would be beneficial to delve deeper into the direct comparison of different classes of living and non-living vaccines to truly assess their potency and suitability for different geographical and epidemiological situations.

## *SALMONELLA* ANTIGENS TO BE INCLUDED IN MULTIVALENT VACCINES

The choice of appropriate combinations of immunogenic and protective antigens would be essential to achieve the maximum strain coverage in a multivalent *Salmonella* vaccine.

**Fig 2:**
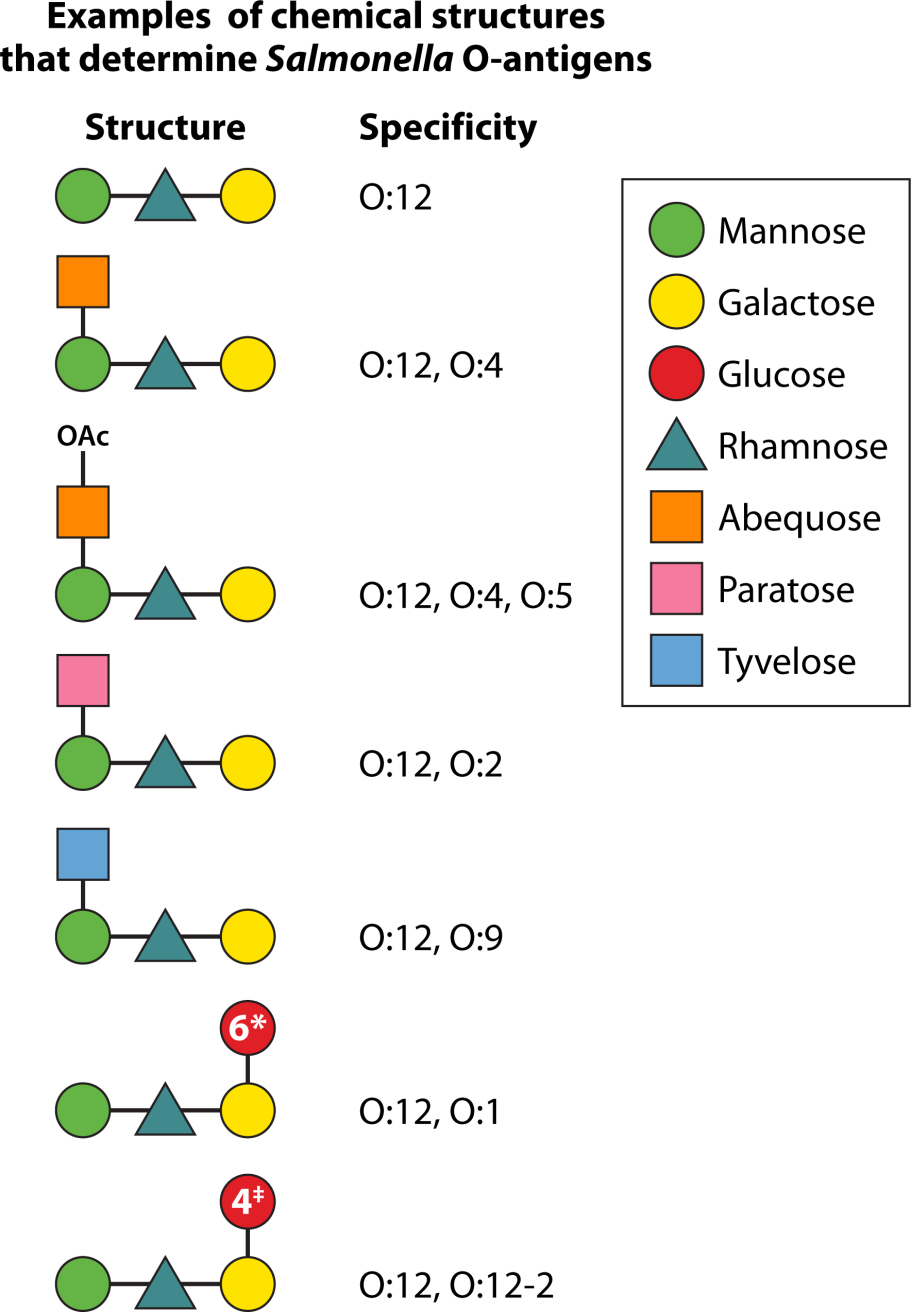
Examples of *Salmonella* O-antigen structures corresponding to antigenic epitopes and indicated with numbers.*1→6 glucosylation of galactose confers O:1 specificity; ^‡^1→4 glucosylation of galactose confers O:12-2 specificity.

The O-polysaccharide antigen is the outermost part of the lipopolysaccharide molecule protruding from the bacterial surface. It consists of repeated sugar units with a backbone shared by many *Salmonella* serovars and specific sugars that define more restricted groups and create immunodominant, serovar-specific determinants. Different structures corresponding to antigenic epitopes are indicated with numbers (64) (Fig. 2). For example, serovar Enteritidis expresses O:1,9,12, and serovar Typhi is O:9,12, with the two serovars sharing the immunodominant O:9 antigen. Serovar Typhimurium expresses O:1,4,[5],12, with O:4 being immunodominant, while serovar Paratyphi A expresses O:1,2,12, with O:2 as the immunodominant antigen. In some cases, 1→4 glucosylation of galactose residues can confer O:12-2 specificity in some serovars (e.g., Typhimurium and Enteritidis). Antibody responses against the O-antigen have been shown to be protective in preclinical models (44, 65–67). Evidence from preclinical studies indicates that antibody-mediated protection and its serovar specificity are mainly due to the immunodominant O-antigens (e.g. O:4, O:9), with shared O-antigenic determinants (e.g., O:12) being insufficient for full cross-protection (68–70). Therefore, inclusion of several O-antigen structures containing the immunodominant O specificity of the desired target strains, mainly O:4, O:9, and O:2, is desirable in the design of broadly protective *Salmonella* vaccines.

Serovar Typhi and some isolates of serovars Paratyphi C and Dublin express the Vi polysaccharide antigen on their surface. Vi is a polymer of α-d-(1–4)-linked *N*-acetylgalactosaminuronate (71). Several Vi-protein conjugates are currently licensed for use in humans and have proven to be safe and effective vaccines for the prevention of typhoid fever (20). Vi is a virulence determinant for the bacteria due to its antiphagocytic properties and its ability to reduce complement activation and deposition on the bacterial surface. Vi also reduces the level of inflammatory responses triggered by the bacteria hindering the stimulation of host pattern recognition Toll-like receptors by other *Salmonella* surface structures (72–74). Vi regulation *in vivo* is complex, and bacteria need to maintain an optimal level of Vi expression in each phase of the infection to maximize infectivity and avoid immune responses. Vi expression is downregulated *in vivo* in the spleen and liver, with most bacteria gradually becoming Vi-negative and therefore no longer expressing this antigen target as the infection progresses (75). There is also a danger that large-scale immunization with vaccines containing the Vi antigen could favor the emergence of Vi-negative isolates of serovar Typhi. These are known to circulate at low levels in endemic areas, are infectious, and are capable of causing disease (74, 76). Therefore, an appropriate combination of Vi and O-antigens would be desirable for inclusion in *Salmonella* multivalent vaccines. For example, a vaccine capable of eliciting both anti-O:9 and anti-Vi antibodies would be able to target bacteria of serovars Typhi and Dublin even if these downregulated Vi in the tissues and would also target Vi^-^ field strains. Such a vaccine would also target serovar Enteritidis.

Many other cell surface and intracellular *Salmonella* antigens, including lipoproteins, porins, heat-shock proteins, secreted proteins, and flagella, have been shown to be immunogenic in animal models and in humans (77–79). The full potential of these immunogenic antigens as vaccine targets remains to be established. Evidence has been provided for a potential protective role of several porins (80–83) and for the secreted protein SseB (78). Furthermore, OMV-based vaccines contain a multitude of *Salmonella* proteins whose protective and cross-protective potential within this delivery system has not been fully studied. A multivalent vaccine containing also immunogenic *Salmonella* proteins that are highly conserved and accessible to antibodies within the outer membrane (83) would be desirable for several reasons. Firstly, these proteins would generate *Salmonella*-specific T and B cell immunological memory that would be broadly cross-reactive between serovars and would be reactivated by the pathogens at the time of infection (54, 80, 81, 84–86). Secondly, antibodies against surface proteins could contribute to the overall protective ability of the immune response (86–89). Thirdly, T cell immunity against *Salmonella* proteins would contribute to enhancing the antibody response and shaping the isotype profile (54). The core region of LPS molecules is highly conserved across various *Salmonella* serovars. While some promising preclinical studies show that the core region may function as a protective antigen in *Salmonella* challenge models, the extent to which antibodies targeting this region can mediate cross-protection following vaccination in humans remains uncertain and requires further investigation (90).

## IMMUNOLOGICAL QUALITATIVE REQUIREMENTS OF THE PROTECTIVE RESPONSE

The development of modern multivalent *Salmonella* vaccines is progressively focusing on combinations of non-living components (20), implying reliance on antibody responses as the main effector mechanisms. Therefore, it will be essential to focus on the development of multivalent vaccines that produce optimized humoral responses with the highest possible level of functional activity. The engenderment of antibodies to *Salmonella* and their qualitative profile are underpinned by a complex crosstalk between T cells and B cells (54, 91, 92), which can be modulated by appropriate choices of vaccine platforms, antigens, and adjuvants.

The isotype profile in relation to antigen specificity significantly influences the efficacy and the effector mechanisms of the antibody response. For example, anti-O:4 IgG2b and IgG2a were shown to have a higher protective ability than IgA and IgG1 to reduce bacterial loads in mouse tissues. Conversely, when considering antibodies against OmpD, IgG1 was shown to be more protective than IgG2a (82). Studies using human chimeric immunoglobulins with identical variable regions (antigen binding region), but constant regions of different isotypes, have revealed the superiority of IgG3 followed by IgG1 and IgG4 in enhancing bacterial uptake by phagocytes compared to IgG2 (93, 94). This appears to parallel the observation that sera from HIV-infected individuals fail to kill *Salmonella* due to inhibitory effects of anti-LPS IgG2 and IgA (89, 95). The mouse and human data outlined above indicate a relationship between the ability to bind activating Fc receptors (i.e., FcγRI) and to activate complement and the relative efficacy of different isotypes. Therefore, individual IgG subclasses to each individual antigen can provide different levels of protection, and this may need to be considered in the design of multiantigen vaccines and delivery platforms.

In summary, several target antigens can be considered for inclusion in *Salmonella* multivalent vaccines to achieve broad coverage of epidemiologically relevant iNTS serovars and to provide immunity against enteric fevers. The qualitative optimization of the immune response is of paramount importance, and this needs to be fine-tuned via the wise choice of antigens and delivery platforms. Multiple O-antigens appear to be key candidates together with the Vi surface polysaccharide. Carrier protein antigens conjugated to the sugar moieties are also essential to ensure the recruitment of T cell help, ultimately leading to improved and isotype-switched antibody responses to polysaccharides. The inclusion of *Salmonella* proteins would most likely be beneficial, as some of these can be protective targets. Furthermore, *Salmonella*-specific T cell memory to be generated during immunization would ensure a more robust recall of secondary immunity upon reinfection.

## VACCINES IN DEVELOPMENT AND TECHNICAL FEASIBILITY FOR COMBINATIONS OF ANTIGENS

The principal vaccines under development at the preclinical and clinical level are described below alongside considerations on the technical and clinical feasibility of combination vaccines.

Several licensed vaccines are available for typhoid fever (Fig. 1) (20). Ty21a is a live attenuated vaccine derived from a chemically mutagenized *S*. Typhi Ty2 strain but, notably, does not express the Vi antigen (96, 97). Despite limitations, such as reduced seroconversion in young children, thermal instability, and reliance on a robust cold chain, Ty21a shows promise for inducing T cell immunity and cross-protection against non-Typhi serovars, including clinical and *in vitro* evidence for cross immunity against *S*. Paratyphi A and B (98–100). The humoral immune response induced by Ty21a predominantly targets O:9, shared by *S*. Enteritidis and *S*. Typhi, suggesting potential utility against iNTS (101). The Vi polysaccharide (e.g., Typhim Vi, Typherix, Typbar Vi, Typho Vi) is a T cell-independent antigen and is licensed for use in children over two years. A single dose of Vi achieves a comparable three-year efficacy of 55% to three doses of Ty21a (102, 103). The first licensed typhoid conjugate vaccine (TCV), Typbar TCV, consists of the Vi capsular polysaccharide chemically linked to tetanus toxoid (TT) as the carrier protein. Developed by Bharat Biotech International Ltd., India, it was licensed by the Drugs Controller General of India (DCGI) in 2013 for use in India. Following its demonstrated efficacy in a controlled human infection model (CHIM) (61), the vaccine was prequalified by WHO in 2018 (104). Post-licensure studies confirmed vaccine efficacy in the field (62, 105, 106) including against extensively drug-resistant (XDR) typhoid (107). Vi-CRM_197_ TCV (TYPHIBEV), consisting of Vi conjugated to the nontoxic mutant of diphtheria toxin CRM_197_, was licensed and WHO prequalified in 2020 (108). In 2024, two additional conjugates have been WHO prequalified: SKYTyphoid (SK Bioscience Co., Ltd.) and ZyVac (Zydus Lifesciences Limited), consisting of Vi polysaccharide linked to DT and TT, respectively.

Currently, no licensed vaccines exist against *S*. Paratyphi A and iNTS, although many are in development (Fig. 1). Bivalent vaccines against both *S*. Typhi and *S*. Paratyphi A are undergoing clinical trials. Biological E, in partnership with GSK Vaccines Institute for Global Health, has combined TYPHIBEV TCV with an O:2-CRM_197_ *S*. Paratyphi A component (109), which was recently tested in a phase 1 clinical trial in Belgium (NCT05613205). The Serum Institute of India is also developing a bivalent vaccine combining Vi-TT and O:2-DT conjugates. This has shown a good safety profile and >90% seroconversion rates for anti-Vi IgG, anti-Vi IgA, anti-*S*. Paratyphi A LPS IgG, and SBA against Paratyphi A (110). Other bivalent vaccines are in preclinical stages. SK Bioscience and IVI have combined Vi-DT (licensed as SKYTyphoid) with O:2-DT. Bharat Biotech, in collaboration with the University of Maryland, has combined a live attenuated *S*. Paratyphi A vaccine (CVD 1902) with a live attenuated typhoid vaccine, CVD 909, resulting in an oral bivalent enteric fever candidate vaccine (20). GSK Vaccines Institute for Global Health is developing a bivalent vaccine against iNTS, using the GMMA (Generalized Modules for Membrane Antigens) technology to deliver *S*. Typhimurium and *S*. Enteritidis O-antigens. GMMA are OMVs naturally released from Gram-negative bacteria that have been genetically modified to destabilize the linkage between the bacterial inner membrane and outer membrane, leading to increased vesicle release. GMMA expose the immune system to *Salmonella* outer membrane and periplasmic proteins, in addition to the O-antigen (65). The ability of this vaccine to elicit strong anti-O-antigen–specific IgG response with bactericidal activity and contribution of proteins to protection was shown in preclinical models (65, 111). This vaccine has recently completed a phase 1 trial in the UK, confirming the ability of the candidate vaccine to induce anti-OAg IgG with bactericidal activity (ISRCTN51750695) (112, 113). Phase 1 and 2 trials are currently ongoing in Kenya (PACTR202310834458532) and Ghana (NCT06213506), respectively. The iNTS-GMMA vaccine has also been combined with Vi-CRM_197_ in a trivalent formulation, demonstrating for the first time the feasibility of combining GMMA with the glycoconjugate technology. The vaccine has recently been tested in a phase 1/2 clinical trial in Belgium and Malawi (NCT05480800).

The University of Maryland and Bharat Biotech have developed a trivalent vaccine by combining Typbar TCV with O:4 and O:9 antigens linked to the phase 1 flagellin antigens of *S*. Typhimurium and *S*. Enteritidis, respectively. Rabbits immunized with the trivalent typhoid-iNTS glycoconjugate vaccine produced serum IgG antibodies to all three polysaccharides, with bactericidal activity *in vitro*. The immune serum protected mice after passive transfer against challenge with virulent iNTS Malian blood isolates (114). The vaccine then progressed to two phase 1 studies in the United States (NCT03981952; NCT05525546) (115), and more recently in an age-descending trial in three African sites, where the lowest age group consists of 12- to 14-week-old infants (NCT05784701). SK Bioscience, in partnership with IVI, is also developing a trivalent iNTS/typhoid vaccine that merges the licensed Vi-DT with O:4 and O:9 combined with the same carrier protein (20).

All technologies currently used for the most advanced *Salmonella* vaccines in development can support the development of four-component vaccines. Scientists at Boston Children’s Hospital (BCH) have worked on the preclinical development of a pioneering quadrivalent *Salmonella* vaccine, potentially covering all four major invasive *Salmonella* serotypes, using the MAPS technology (116). The vaccine contains Vi from *S*. Typhi, O-antigens from *S*. Paratyphi A, *S*. Enteritidis, and *S*. Typhimurium, alongside the *Salmonella*-specific protein SseB. The vaccines have been shown to elicit robust and functional antibody responses to each of the components in animal models.

An additional approach for the design of multivalent vaccines is to exploit the flexibility of the OMV/GMMA platform not only for the combination of different vesicles in the same formulation (65, 117), but also for the expression or conjugation of multiple heterologous proteins and polysaccharide antigens on the surface of the same vesicle. For example, the *S*. Typhi Vi antigen can be displayed on the surface of *S*. Paratyphi A GMMA. The resulting GMMA constructs induce a strong functional antibody response against both Vi and O:2 in mice (118). Furthermore, the use of appropriate promoters can enable the modulation and maximization of antigen expression, thus achieving optimal combination of protective antigens at high levels on the same vesicle (119–121). In theory, pathogen-specific proteins can be used as carriers also through traditional conjugation, but this may require use of appropriate selective chemistries to preserve protein antigenicity; moreover, not all proteins are equally effective as carriers for glycoconjugates (122).

## ANALYTICAL METHODS FOR MULTIVALENT VACCINES

Despite advancements in the field of multivalent *Salmonella* vaccines, challenges persist in developing accurate analytical methods to monitor higher valency combinations. It is important to develop robust analytical methods to quantify and monitor integrity and purity of each active antigen over time, verify compatibility among different components, ensure stability, and demonstrate no negative immunological interference in the target population.

*Salmonella* O-antigens of different serovars share many sugars in their composition. Therefore, appropriate hydrolysis and chromatographic conditions will be needed to quantify those monosaccharides that differentiate them (e.g., paratose, tyvelose, and abequose). Alternatively, functional monoclonal antibodies could be used in immune assays with the final aim to distinguish and quantify each active component and possibly also to verify *in vitro* potency (123, 124). Analytics used to assess size and integrity of the final formulation (e.g., Size Exclusion High Performance Liquid Chromatography [HPLC-SEC], Dynamic Light Scattering [DLS], Size Exclusion Chromatography with Multi-Angle Static Light Scattering [SEC-MALS]) will need to be adjusted and are likely to provide information on the overall formulation. Accelerated stability studies can help to identify potential mechanisms of degradation and evaluate more appropriate analytical methods to check them. Analytics such as NMR spectroscopy can be used to monitor the structural integrity of each component (e.g., O-acetylation level). The methods identified for vaccine release will also be used to check stability. When combining multiple components in a new formulation, it is critical to monitor the compatibility of all components and to identify optimal buffer and adjuvants, paying attention that the presence of novel ingredients does not accelerate or modify certain instability patterns. Studies to look at the ability of each component to elicit a strong and functional immune response, not inferior to that elicited by its corresponding mono-component versions, need to be performed in different animal models before proceeding to clinical studies.

## REGULATORY PATHWAY FOR *SALMONELLA* COMBINATION VACCINES

Potential challenges for multivalent vaccines relate to the complex, lengthy, and expensive clinical development pathway. Licensure of novel combination *Salmonella* vaccines can be simplified by the inclusion of already licensed TCVs. *Salmonella* combination vaccines that are currently in clinical trials can be broadly divided into bivalent (*S*. Typhi/Paratyphi A) enteric fever vaccines for South/Southeast Asia and bivalent (*S*. Typhimurium/Enteritidis) or trivalent (*S*. Typhi/Typhimurium/Enteritidis) typhoid/iNTS vaccines for Africa. In the case of combinations with TCV, it is necessary to demonstrate the immunological non-inferiority of the typhoid component compared to licensed TCVs. For the *S*. Paratyphi A component, which lacks a Correlate of Protection (CoP), the suggested licensure pathway includes demonstrating protective efficacy in a CHIM study in adults, followed by confirmation of equivalent immune responses in immunogenicity field trials in target populations. For iNTS, a phase 3 efficacy trial in the target population appears feasible. A four-component vaccine could eventually gain licensure based on immuno-bridging (showing no immune inferiority of each component with respect to lower valency formulations), with developers committed to confirm vaccine effectiveness through post-approval studies. There is a possibility that combination vaccines may be less effective than vaccines administered separately, as one of the potential risks of multivalent vaccines could be negative immunological interference. On the other hand, a combination of multiple antigens may broaden the strain coverage of the vaccines by eliciting antibodies against protein and polysaccharide epitopes that are shared by many *Salmonella* serovars. These issues will become clearer with future improvement of our knowledge of vaccine-mediated immunity to *Salmonella* in humans and with a better understanding of correlates of protection (125).

## IMMUNOLOGICAL READOUTS IN CLINICAL TRIALS

Immunological readouts used in clinical trials have focused on the analysis of humoral responses. In approximately 70 clinical trials related to *Salmonella* vaccine development, the primary immunological readout has been the level of anti-Vi and/or anti-O-antigen–specific IgG, while anti-protein antibodies, such as anti-FliC or anti-GMMA, have generally been considered secondary readouts. The anti-Vi IgG titer has been the most frequently used immunoassay. However, an immunological IgG threshold of anti-Vi (or other antigens) antibody levels that correlates with protection has not yet been fully established (126).

Better standardization of immunoassays remains a pressing issue, given the need to compare results between different laboratories and clinical studies. Currently, the only international standard available is for the measurement of the anti-Vi immune response (127). The adoption of anti-Vi international standards allows comparison of results of various studies and the use of different immunoassays (127, 128). Developing standards for antigens other than Vi (i.e., O-antigens and in some cases proteins) represents a crucial challenge, given that many combination vaccines will target these antigens.

In addition to measuring quantitative antibody levels, assessing their functional activity is critical. Complement-mediated serum bactericidal activity (SBA), either with conventional or luminescent-based readouts, and opsonophagocytic killing (OPK) are often taken as a proxy of immunological protection against *Salmonella* in clinical trials and/or in sero-epidemiological studies (113, 129–131) (https://wellcomeopenresearch.org/articles/8-27/v1). The use of functional assays not only offers the possibility to confirm the ability of antibodies to kill the pathogen expressing the vaccine antigen target, but also allows gaining preliminary insights on the breadth of vaccine protection, by probing sera of vaccinees against a panel of clinically relevant isolates expressing antigens with different chemical features (i.e., different level of glycosylation or O-acetylation) (132, 133).

More work is needed to conclusively ascertain the robustness of the above-mentioned functional antibody assays as true mechanisms and correlates of protection. For example, *in vitro* studies raise doubts about the relevance of SBA as a host resistance mechanism against invasive *Salmonella* because the bacteria can establish intracellular infection before being killed by antibodies and complement (i.e., SBA) (134). Similarly, although antibody- and complement-dependent phagocytosis, followed by activation of reactive oxygen–mediated intracellular killing, appears to be a primary mechanism of *Salmonella* killing in human blood, its true relevance as an *in vivo* protective mechanism remains to be fully established (49).

Other features of the antibody response, such as avidity and antigen-binding strength, can be taken into consideration toward a more comprehensive approach to establish and monitor vaccine-mediated correlates of protection. The use of “systems serology” will progressively integrate more comprehensive analyses of antibody features and functions (135). Multiple parameters, including binding specificity, isotype/subtype classification, avidity, glycosylation profiles, immune cell activation, and Fc receptor engagement, will progressively be integrated into complex analyses that will provide improved correlations between immune responses and vaccination outcomes. For example, using systems serology, it has recently been proposed that anti-Vi IgA levels and avidity correlate with protection against *S*. Typhi infection, whereas higher increases in Vi IgG responses were associated with reduced disease severity. Targeted antibody-mediated functional responses, particularly neutrophil phagocytosis, were also identified as important components of the protective signature of Vi vaccines (136). Integrated systems serology will offer the possibility to globally analyze multiple parameters relative to a broad range of antigens. Therefore, it is feasible that these approaches will be very useful for monitoring the responses to multi-antigen combination *Salmonella* vaccines and for evaluating correlates of protection to multiple serovars simultaneously.

Broadening the number of parameters to be monitored during clinical trials will be useful for a better assessment of responses and correlates of vaccine-induced protection to any vaccine, including *Salmonella* combination vaccines. In addition to the assessment of antibody responses in sera, immunological studies in clinical trials will benefit from the analysis of antigen-specific memory B cell and T cell responses pre- and post-vaccination (137–141). In the context of multivalent *Salmonella* vaccines, the whole range of immunological parameters will need to be considered in relation to each individual antigen, also taking into consideration cross-reactivity between target strains due to shared antigens. This is significant for B cell responses, as many *Salmonella* serovars share common polysaccharide determinants, enabling potential cross-reactive antibody production. It is even more relevant for T cell immunity, where the high degree of protein similarity among *Salmonella* serovars suggests a strong likelihood of cross-reactive T cell responses across different serovars. Controlled human infection model (CHIM) studies, which involve the deliberate exposure of healthy human volunteers to a controlled dose of an infectious agent, are used to preliminarily test a vaccine’s ability to confer protection and to examine correlations with immune markers. CHIM has been developed for *S*. Typhi, *S*. Paratyphi A, and *S*. Typhimurium (142–145). CHIM studies of infection with heterologous serovars could help to understand cross-protection in the context of *Salmonella* vaccines.

## CONCLUSIONS

Recent progress in our knowledge of the within-host immunity and pathogenesis of *Salmonella,* combined with a better understanding of the epidemiology of disease, is leading to rational advancements in vaccine design. The geographical overlap in distribution of different serovars of *Salmonella* makes multivalent vaccines an increasingly attractive option to achieve protective immunity toward a broad range of *Salmonella*. Several multicomponent vaccines utilizing different platforms are currently in preclinical development and/or progressing into the clinical phase. The possibility to engineer vaccines consisting of fewer components, but still expressing a high number of protective antigens, thus retaining breadth of protection against multiple serovars, is very attractive. Although this may pose surmountable challenges at the stages of development and licensure, it would provide more refined and cost-effective tools toward a rapid reduction of disease burden, especially in those areas where different *Salmonella* diseases coexist.
